# The bleaching efficacy of carbamide peroxide gels containing potassium nitrate desensitizer

**DOI:** 10.4317/jced.56917

**Published:** 2020-07-01

**Authors:** Adriana-Osten Costacurta, Carolina-Elisa-Pereira Borges, Camila Centenaro, Gisele-Maria Correr, Marina-da Rosa Kaizer, Carla-Castiglia Gonzaga

**Affiliations:** 1 DDS, MS. Graduate Student, School of Health Sciences, Graduate Program in Dentistry, Universidade Positivo, Curitiba, PR, Brazil; 2 Undergraduate Student, School of Health Sciences, Graduate Program in Dentistry, Universidade Positivo, Curitiba, PR, Brazil; 3 DDS. Graduate Student, School of Health Sciences, Graduate Program in Dentistry, Universidade Positivo, Curitiba, PR, Brazil; 4 DDS, MS, PhD. Graduate Student, School of Health Sciences, Graduate Program in Dentistry, Universidade Positivo, Curitiba, PR, Brazil; 5 DDS, PhD. Professor, School of Health Sciences, Graduate Program in Dentistry, Universidade Positivo, Curitiba, PR, Brazil

## Abstract

**Background:**

To evaluate the bleaching efficacy of at-home carbamide peroxide (CP) gels in two concentrations, containing or not a desensitizing agent.

**Material and Methods:**

Forty incisors were divided into four groups (n=10), according to gel concentrations (10% or 22%), and presence or not of 3% potassium nitrate in the gel. A thin layer of gel was applied to the buccal surface of each tooth for 2h/day for 4 weeks. Bleaching efficacy was measured using a spectrophotometer, and ∆E*ab, ∆E00 and ∆WID were calculated. Measurements were performed at baseline, 7, 14, 21, 28, and 35 days following the first gel application. Data were analyzed by two-way RM-ANOVA and Tukey’s test (α=0.05).

**Results:**

Regarding gel concentration or potassium nitrate inclusion, both gels resulted in color change above the perceptibility thresholds, which were similar between gels. Regarding time, significant differences were observed between color change values at 7 days and other time periods. ∆WID ranged from 3.8 to 9.6. Significant moderate to strong positive correlation was observed among the parameters.

**Conclusions:**

Nor the CP concentration, neither the inclusion of potassium nitrate in the gel, had influence on bleaching efficacy. All gels were effective and showed good results from the first weeks’ application.

** Key words:**Tooth bleaching, carbamide peroxide, desensitizer, potassium nitrate, color.

## Introduction

The demand for esthetic treatments is increasing among patients, and dental bleaching is a frequent esthetic procedure performed in clinical practice. At-home bleaching technique is effective, providing patient satisfaction with minimal long-term side effects (1). Hydrogen peroxide is the most widely used bleaching agent, which can be applied directly to the tooth or produced locally by chemical reactions using sodium perborate or carbamide peroxide as precursors (2). For at-home bleaching, agents with relatively low concentrations of peroxide are preferred, due to improved safety and reduced risk to tooth sensitivity. Literature reports that severe tooth sensitivity may occur during and after bleaching as a response to the use of gels with high peroxide concentrations (3-5).

Hydrogen peroxide has low molecular weight, and ability to diffuse through the enamel and dentin and potentially reach the pulp, which may explain the frequent patient reports of tooth sensitivity during treatment (6). This short-term side effect is reported by 15 to 65% of patients using 10% carbamide peroxide gels (7). Studies have proposed several alternatives to minimize tooth sensitivity, including reduction in gel concentration, decrease in time and frequency of use (8), and use of various desensitizing agents, such as potassium nitrate, before or after bleaching procedures, or included in the bleaching gels (9-13). Potassium nitrate is able to reduce tooth sensitivity by decreasing the ability of dental pulp nerve fibers to repolarize after initial depolarization due to pain (11). Nevertheless, it is still unclear whether the presence of potassium nitrate in the gel can affect its bleaching efficacy for at-home bleaching.

Thus, this study aimed to evaluate the bleaching efficacy during the course of, and short-term after an at-home bleaching protocol using carbamide peroxide in two concentrations (10% and 22%), with or without the presence of a desensitizing agent (3% potassium nitrate) in the gels. The null hypotheses of the study were: 1) carbamide peroxide concentration would not influence the efficacy of at-home bleaching, 2) presence of potassium nitrate in the gel would not influence the efficacy of at-home bleaching.

## Material and Methods

Forty bovine incisors free of enamel defects or evident discoloration were firstly disinfected in chloramine solution (1%) for 24h. Then the teeth were stored refrigerated (4°C) immersed in distilled water, which was changed weekly, until the beginning of the experiments. Prior to bleaching procedures, the teeth were cleaned with pumice and randomly divided into four groups (n = 10), according to the carbamide peroxide concentration (10% or 22%), and presence or not of 3% potassium nitrate (desensitizing agent) in the bleaching gel. The experimental gels were produced by a pharmaceutical laboratory and stored refrigerated during the course of the experiments.

To standardize the area where the color readings were carried out, a 6 x 6 mm squared shallow groove was created with a spherical diamond bur (FG1012, KG Sorensen, Cotia, SP, Brazil) on the center of the buccal surface of each tooth. The baseline color readings were conducted immediately before the first bleaching application.

Before gel application, the teeth were gently dried with absorbent paper to remove excess moisture. An even 2 mm thick layer of bleaching gel was applied to the buccal enamel surface. The teeth were kept in 100% humidity at 37°C for 2 hours in contact with the bleaching gel. This procedure was repeated daily for 4 weeks. After each application, the teeth were washed thoroughly under tap water for complete removal of the gel, followed by storage immersed in distilled water at 37°C. After the last application of the bleaching gel, the specimens were treated with topic fluoride gel.

The bleaching efficacy of the four gels was evaluated by using an objective color measurement with a spectrophotometer (EasyShade Advance, Vita Zahnfabrik, Bad Säckingen, Germany). All color readings were performed within the previously defined area on the center of the buccal surface of the teeth. A research laboratory was chosen for the procedures, having standard temperature, humidity, and illumination conditions. Color readings were conducted at baseline (prior to bleaching), and then repeated at 7, 14, 21, 28, and 35 days after the first bleaching application.

For the spectrophotometric evaluation, the specimens were gently dried with absorbent paper, and placed on a flat surface with a standard white background. The spectrophotometer was calibrated before the readings, and was always positioned at a 90° angle to the surface. The CIEL* a* b* tridimensional color space was used, where L* indicates luminosity axis (L*=0 is black and L*=100 is white), a* represents the greenness (-a*) and redness (+a*) axis, and b* represents the blueness (-b*) and yellowness +b* axis.(14) 

**Figure 1:**
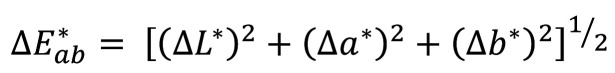
Formula.

**Figure 2:**
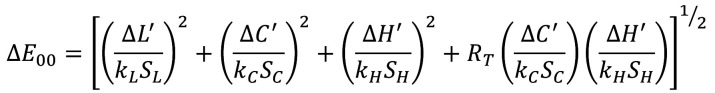
Formula.

Color stability was assessed by calculating the color difference between each time interval and baseline. Both the equations ∆E*ab (Eq. 1) and the ∆E00 (Eq. 2) were used in this study (14): (Fig. 1).

where ∆L*, ∆a*, and ∆b* are the difference between a pair of color coordinates measure at baseline and each time interval, (Fig. 2).

where ∆L’, ∆C’, and ∆H’ are the differences in lightness, chroma, and hue for a pair of color measurements (baseline and each time interval) in CIEDE2000. RT is a rotation function that accounts for the interaction between chroma and hue differences in the blue region. The weighting functions SL, SC, and SH adjust the total color difference for variation in the location of the color difference pair at the L*, a*, and b* coordinates, and the parametric factors kL, kC, and kH are correction terms for experimental conditions. In the present study, kL, kC, and kH were set to 1.

The 50:50% acceptability thresholds for CIEL* a* b* (∆E*ab) and CIEDE2000 (∆E00) were 2.7 and 1.8, respectively (15).

**Figure 3:**
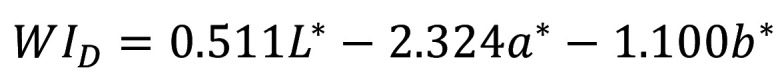
Formula.

The CIEL* a* b*color space-based whitening index (WID) was calculated for each assessment time according to the formula (16): (Fig. 3).

The ∆WID was calculated by the difference between the indices at baseline and each time interval (∆WID=|∆WID2-∆WID1|). The whiteness difference threshold for acceptable bleaching effect was 2.60 WID units (17).

The effectiveness of tooth whitening was determined based on the interpretation of visual thresholds, adopting a classification system from 1 to 5, derived from research results on PT (perceptibility threshold) and AT (acceptability threshold) (18). In this system, index 1 corresponds to ineffective bleaching (<PT) and index 5 to excellent bleaching efficiency (>AT × 3).

The color difference data were analyzed by three-way RM-ANOVA and Tukey’s test. The correlations among ∆E*ab, ∆E00 and ∆WID were determined by Pearson’s correlation coefficient. All analyses were performed with a 5% significance level.

## Results

The means and standard deviations for ∆E*ab, ∆E00, ∆WID are shown in Tables 1 to 3, respectively.

**Table 1:**
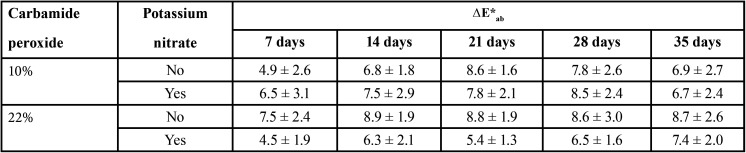
Means and standard deviations of ∆E*ab for the carbamide peroxide gels as a function of concentration and inclusion of potassium nitrate.

**Table 2:**
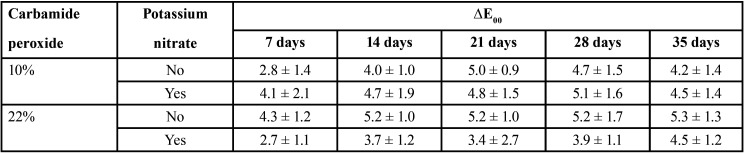
Means and standard deviations of ∆E00 for the carbamide peroxide gels as a function of concentration and inclusion of potassium nitrate.

**Table 3:**
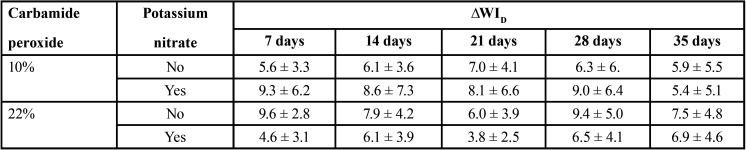
Means and standard deviations of ∆WID for the carbamide peroxide gels as a function of concentration and inclusion of potassium nitrate.

For ∆E*ab, statistically significant differences were observed for inclusion of potassium nitrate (*p* = 0.04) and time (*p* <0.001), and for the double interactions of concentration of gel*potassium nitrate inclusion (*p* = 0.007) and concentration of gel*time (*p* = 0.04). Gel concentration (*p* = 0.91) and the other double and triple interactions were not statistically significant (*p* > 0.05).

With regard to gel concentration, pooled mean ∆E*ab values for 10% (7.2 ± 2.6) were similar to those for 22% (7.3 ± 2.5). For the inclusion of potassium nitrate, pooled mean ∆E*ab data showed that gels with potassium nitrate (6.7 ± 2.4) resulted in statistically lower ∆E*ab values than those without potassium nitrate (7.8 ± 2.6). Regarding time, pooled mean ∆E*ab values were: 7 days (5.9 ± 2.7), 14 days (7.4 ± 2.3), 21 days (7.7 ± 2.1), 28 days (7.9 ± 2.5), and 35 days (7.4 ± 2.5). Significant difference was observed between ∆E*ab values at 7 days and other evaluation time periods, which presented statistically similar ∆E*ab.

For ∆E00, statistically significant differences were observed for time (*p* < 0.001) and for the double interaction of concentration of gel*potassium nitrate inclusion (*p* = 0.001). Gel concentration (*p* = 0.81), potassium nitrate inclusion (*p* = 0.19) and the other double and triple interactions were not statistically significant (*p* > 0.05).

With regard to the gel concentration, pooled mean ∆E00 values for 10% (4.4 ± 1.6) were similar to those for 22% (4.3 ± 1.4). For the inclusion of potassium nitrate, pooled mean ∆E00 data showed that gels with potassium nitrate (4.2 ± 1.5) resulted in statistically similar values than those without potassium nitrate (4.6 ± 1.4). Regarding time, pooled mean ∆E00 values were: 7 days (3.5± 1.6), 14 days (4.4 ± 1.4), 21 days (4.6 ± 1.2), 28 days (4.7 ± 1.5), and 35 days (4.6 ± 1.3). Significant difference was observed between ∆E00 values at 7 days and other evaluation time periods, which were statistically similar.

All groups had ∆E*ab and ∆E00 greater than the 50:50% acceptability thresholds at all evaluated time points. Regardless of gel concentration and presence of potassium nitrate, at-home bleaching was effective in all groups from the first week of application, and best results were attained after 14 days, and maintained in the subsequent times ( Table 1, Table 2).

For ∆WID all individual factors, double and triple interactions were not statistically significant (*p* > 0.05). ∆WID ranged from 3.8 to 9.6, and was above the whiteness difference threshold for accepTable bleaching effect (2.60 units) in all groups.

**Figure 4:**
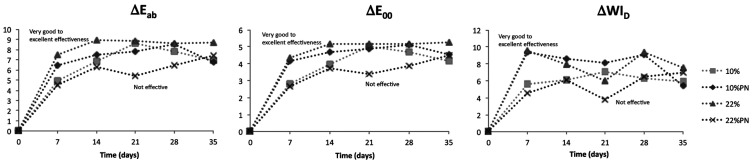
Color change parameters (∆E*ab, ∆E00 and ∆WID) for follow-up times compared to the immediately preceding time for the assessed groups.

Significant strong positive correlation was observed between ∆E*ab and ∆E00 values (R = 0.98, *p* < 0.01). Significant moderate positive correlations were observed between ∆E*ab and ∆WID values (R = 0.60, *p* < 0.01) and between ∆E00 and ∆WID values (R = 0.59, *p* < 0.01), (Fig. 4).

## Discussion

The first hypothesis, that carbamide peroxide concentration would not influence the efficacy of at-home bleaching, was accepted, since gels containing 10% and 22% of carbamide peroxide yielded similar statistical results. Reports have indicated that peroxide concentration and application time are the main factors to determine the efficacy of tooth bleaching (19,20). Gels with higher peroxide concentrations may be more effective, yielding bleaching in a shorter period of times, but may also result in greater side effects (21). However, somewhat contradictory results have also been reported, showing similar, or ever better, bleaching efficacy with gels containing low peroxide concentrations (10% to 20%), when compared to those with concentrations up to 35% to 38% of carbamide or hydrogen peroxide (5,22). The results of the present study are in agreement with other *in vitro* and clinical investigations, showing that gels with low peroxide concentrations are as effective as gels with high concentrations (4,5,22-25).

The second hypothesis, that inclusion of potassium nitrate in the gel would not influence the efficacy of at-home bleaching, was accepted, since gels with and without potassium nitrate resulted in similar bleaching efficacy. A systematic review and meta-analysis (20) evaluated the efficacy of desensitizing agents including potassium nitrate and sodium fluoride on tooth bleaching. Potassium nitrate and/or sodium fluoride reduced the tooth sensitivity, but the review reported inconsistent findings in terms of bleaching efficacy. The discrepancy in results between the studies may be explained by the distinct delivery methods of the desensitizing agents. In that systematic review (20), studies using different desensitizing agents delivery methods were included, such as, (i) application of desensitizing agent on the surfaces of the teeth prior to bleaching; or (ii) desensitizing agent is present as a component of the bleaching gel, as in the present study.

This study used three different parameters (∆E*ab, ∆E00, and ∆WID) to evaluate bleaching efficacy, all based on objective and standardized color readings, using a spectrophotometer to determine CIEL* a* b* coordinates (14). Visual shade matching using Vita Classical and Vita 3D Master shade guides is a common approach in the clinical setting. Nonetheless, this approach is subjective and depends on visual perception and acuity, training and environment. Therefore, instrumental color determination (spectrophotometry) and the use of standardized formulas to calculate color difference should be preferred, enabling detection of minor differences, hardly perceived by the general observer (15,26).

∆E*ab is one of the most used parameters for dental color evaluation (15). However, the use of CIEDE2000 color-difference formula (∆E00) has been suggested, because it is considered more precise and incorporates specific corrections for non-uniformity of CIEL* a* b* color space, a rotation term (RT), and parameters regarding the influence of illuminating and vision conditions in color difference evaluation (14,27). Significant correlation between ∆E*ab and ∆E00 for different shades of composite resins has been reported, with R2 of 0.99 (28). The present study, evaluating bleaching efficacy on teeth, also found a strong significant positive correlation coefficient (R = 0.98) between ∆E*ab and ∆E00. This strong positive correlation is expected, because these two color difference parameters are calculated based on the measured CIEL* a* b* coordinates.

∆E values do not show the direction of the color change, so it is usually not possible to know for sure if the bleaching procedure was effective or not, with increased ∆L*, without analyzing the individual coordinates. In bleaching studies, in addition to color change, it is important to evaluate a specific parameter for determining the whiteness (level of white). Many whiteness indexes have been described, but recently a new customized CIEL* a* b*-based whiteness index for dentistry (WID) was proposed (16). It has been reported that this index showed a better correlation with the visual perception compared to others CIEL* a* b*-based whiteness indexes (16,29). In the present study, ∆WID showed significant bleaching in the first week and the results were maintained thereafter. Thus, ∆WID showed good agreement with ∆E*ab and ∆E00 data, as shown by the moderate significant positive correlations (∆E*ab x ∆WID (R = 0.60) and ∆E00 x ∆WID (R = 0.59)].

In the present study, bleaching effect was detected as early as the first week, and the results remained sTable from 14 to 35 days. In agreement with these findings, most clinical at-home bleaching protocols recommend a treatment period of 2 to 4 weeks, avoiding prolonged treatment periods, since saturation is attained and no major damage to the tooth structure occurs. In order to thoroughly evaluate bleaching efficacy, it is important to consider the changes in delta values for each individual color coordinate, along with the color difference values (∆E*ab or ∆E00). In the present study, ∆L* reveled a positive variation in all groups and times, indicating that the teeth became lighter (higher L* value), with ∆L* ranging from 1.3 (22% with potassium nitrate, 7 days) to 6.2 (10% with potassium nitrate, 28 days). All groups also presented a coherent variation in ∆b*towards negative values, ranging from -1.3 (22% with potassium nitrate, 21 days) to -6.6 (22%, 7 days), indicating less yellowish and more bluish colors.

It can be concluded that the carbamide peroxide concentration and inclusion of potassium nitrate in the gel did not influence at-home bleaching efficacy. All gels evaluated in this study were effective and showed good bleaching results from the first weeks’ application.
